# Acid-specific formaldehyde donor is a potential, dual targeting cancer chemotherapeutic/chemo preventive drug for FANC/BRCA-mutant cancer

**DOI:** 10.1186/s41021-019-0136-5

**Published:** 2019-12-27

**Authors:** John R. Ridpath, Jun Nakamura

**Affiliations:** 1 Department of Environmental Sciences and Engineering, University of North Carolina at Chapel Hill, Chapel Hill, NC USA; 2 Laboratory of Laboratory Animal Science, Graduate School of Life and Environmental Biosciences, Osaka Prefecture University, Izumisano, Osaka, Japan

**Keywords:** Hexamethylenetetramine, Formaldehyde, Fanconi anemia, BRCA, Cancer

## Abstract

**Background:**

Development of chemotherapeutic/preventive drugs that selectively kill cancer - the Holy Grail of cancer research - is a major challenge. A particular difficulty arises when chemotherapeutics and radiation are found to be rather ineffective against quiescent cancer cells in solid tumors. In the limited oxygen condition within a solid tumor, glycolysis induces an acidic environment. In such an environment the compound hexamethylenetetramine (HMTA) will act as a formaldehyde donor. HMTA has been characterized a non-carcinogen in experimental animals and causes no major adverse side-effects in humans. We previously reported that both a chicken B-lymphocyte cell line transformed with an avian leucosis virus and human colon cancer cells deficient in the FANC/BRCA pathway are hypersensitive to formaldehyde. Thus, we assessed the potential usage of HMTA as a chemotherapeutic agent.

**Results:**

The differential cytotoxicity of HMTA was tested using chicken DT40 cells deficient in DNA repair under neutral and acidic conditions. While HMTA is not efficiently hydrolyzed under neutral conditions, all HR-deficient DT40 cells tested were hypersensitive to HMTA at pH 7.3. In contrast, HMTA clearly increased cell toxicity in FANCD2-, BRCA1- and BRCA2- deficient cells under acidic conditions.

**Conclusion:**

Here we show that in vitro experiments showed that at low pH HMTA causes drastic cytotoxicity specifically in cells deficient in the FANC/BRCA pathway. These results strongly suggest that HMTA may be an attractive, dual-targeting chemotherapeutic/preventive drug for the selective delivery of formaldehyde to solid tumors and causes cell death in FANC/BRCA-deficient cells without major adverse effects.

## Introduction

Germ-line mutations in the breast cancer genes *BRCA1* and *BRCA2* result in predisposition to breast and ovarian cancers (*BRCA1*) as well as other cancers (*BRCA2*) [1]. Five to 10 % of breast cancer cases are associated with genetic factors, while 90–95% of cases are considered to be sporadic [2]. In cases where heredity is a contributing factor, *BRCA1* has been found to be mutated at a rate of 40–45%, whereas *BRCA2* is mutated 35–40% [2–4]. Non-mutational deficiencies in BRCA1 can also exist due to down-regulation caused by promoter hypermethylation [5, 6] observed in 30–40% of sporadic breast cancer cases [2]. The BRCA1/FANCS and BRCA2/FANCD1 gene products have been found to be involved in DNA double strand-break (DSB) repair and DNA interstrand cross-links (ICLs) repair by homologous recombination (HR) and Fanconi anemia pathway [7–11]. ICLs are extremely deleterious lesions caused by bi-functional alkylating agents that covalently tether both duplex DNA strands and pose formidable blocks to DNA metabolism [12]. Critical to ICL repair is the formation of a single-stranded DNA intermediate; this explains why HR is necessary in the repair of these lesions [12, 13]. It is therefore not surprising that either BRCA1 or BRCA2 deficient cells have been found to be hypersensitive to ICL-inducing agents such as the chemotherapeutics cisplatin and mitomycin C [14, 15]. Paradoxically, these agents have the potential to induce secondary cancer. A DNA lesion similar to the ICL is the DNA-protein cross-link (DPC). Compared to the ICL, however, the repair mechanism(s) of DPCs have not been well characterized.

We first discovered that HR-deficient DT40 cells, particularly those with a deficiency in FANCD2, BRCA1 and BRCA2 were hypersensitive to formaldehyde, a well-known DPC inducer, at concentrations commonly found in human plasma [16]. We also found human cancer cells deficient in FANCC and FANCG to be hypersensitive to formaldehyde at similar concentrations. The DT40 cells deficient in FANC/BRCA pathways were also sensitive to very high concentrations of acetaldehyde [16]. Considering that HR-deficient cells are hypersensitive to formaldehyde, we hypothesized that a formaldehyde donor-molecule could be an attractive cancer cell-specific therapeutic/preventive agent in these cells.

**Fig. 1:**
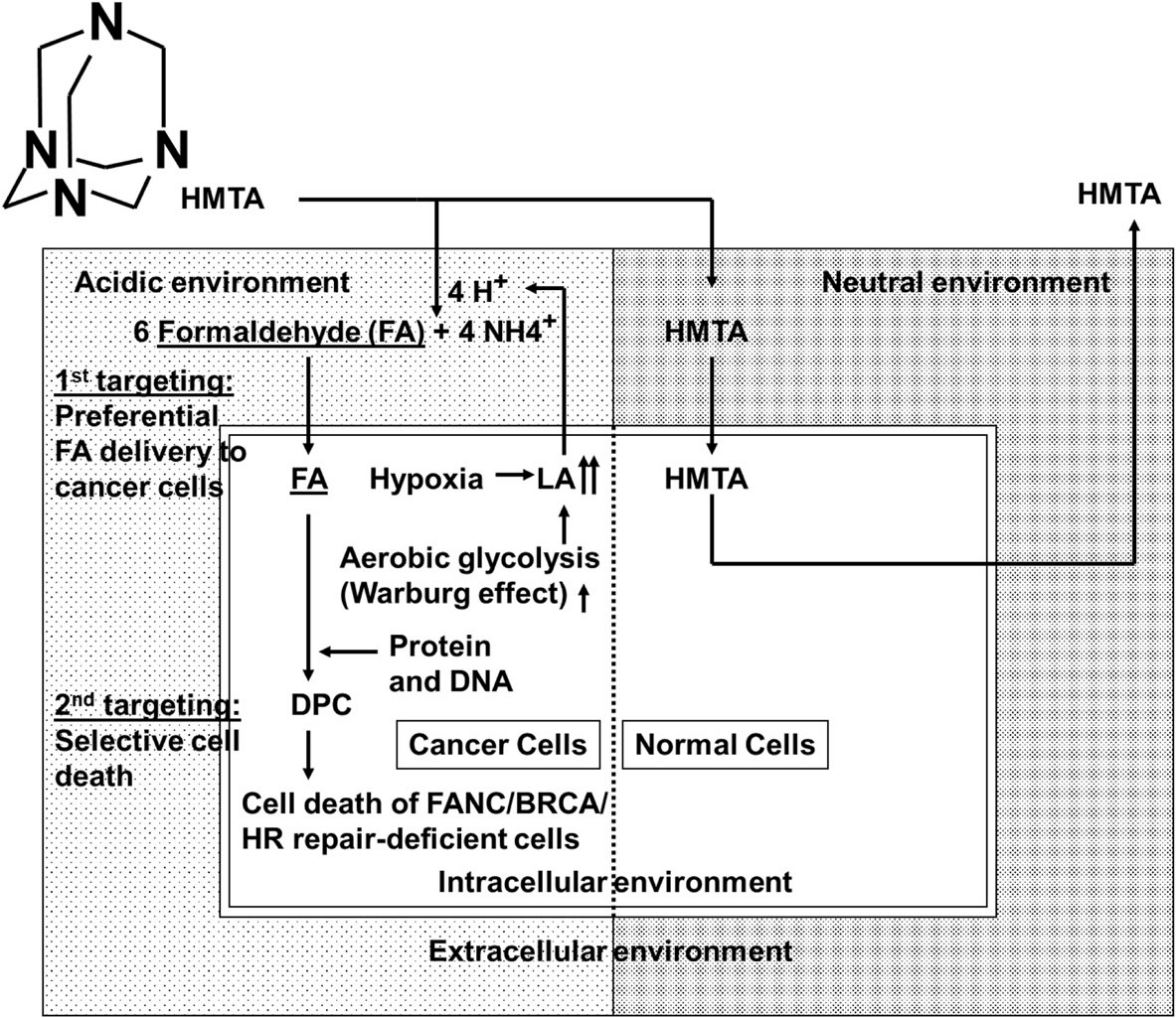
Hypothetical mechanisms by which HMTA causes dual targeting effects on DNA repair-deficient cells. 1st targeting: Preferential formaldehyde delivery to cancer cells. Under the acidic conditions of the extracellular environment of cancer cells, HMTA is preferentially hydrolyzed to 6 molecules of formaldehyde and 4 molecules of ammonium ions. Therefore, formaldehyde preferentially introduces DPCs in cancer cells. 2nd targeting: Selective cell death for cancer cells. Formaldehyde selectively kills FANC/BRCA- and HR-deficient cells probably due to a deficiency in DPC repair. In contrast, normal cells are resistant to HMTA because less formaldehyde is formed in a neutral environment and they are proficient in the repair of formaldehyde induced DNA damage

In a limited oxygen environment, such as that within a solid cancerous tumor, pyruvate generated by glycolysis in the cytoplasm of the cell is preferentially converted into lactic acid by lactate dehydrogenase, which induces a low pH environment. Furthermore, many cancer cells vigorously consume glucose and preferentially produce lactic acid even in the presence of adequate oxygen, a concept known as the Warburg effect [17]. It has been shown that while intracellular pH levels are neutral or alkaline [18], the extracellular pH is acidic [19]. Hexamethylenetetramine (HMTA) is a tertiary amine that becomes hydrolyzed in acidic conditions to generate four molecules of ammonia and six molecules of formaldehyde from one parent molecule (Fig. 1) [20]. Therefore, due to the extracellular acidic conditions within solid tumors it would be expected that HMTA would dissociate to release formaldehyde within the tumor mass. HMTA has been used as an antiseptic for treatment of urinary tract infections. It has also been used as a prophylactic agent against recurrent acute cystitis, and as an antibacterial preservative in food and cosmetics. It is believed to have low toxicity and has been characterized as a non-carcinogen in animal studies [21]. In the oncology studies, HMTA has been experimentally applied to mice bearing squamous cell carcinoma. Hypoxic conditions in tumors induce cell quiescence leading to resistance to radiation and chemotherapeutic agents. In the limited oxygen condition existing in a solid tumor, glycolysis induces an acidic environment, a phenomenon termed the Warburg Effect [22]. Previous studies conducted by Masunaga, et al.*,* demonstrated chemosensitization with methenamine [23–26] . In the present study, we addressed the potential usage of HMTA, an acid-specific formaldehyde donor, to act as a dual-targeting cancer chemotherapeutic agent without causing major toxicity.

## Materials and methods

### Materials

Fetal bovine serum, HMTA, lactic acid (LA), 2,3-bis (2-methoxy-4-nitro-5-sulfophenyl)-2H-tetrazolium-5-carboxanilide (XTT), and 1-methoxy-5-methylphenazinium methyl sulfate (1-methoxy PMS) were obtained from Sigma. RPMI-1640 culture medium, chicken serum and penicillin/streptomycin were obtained from Invitrogen.

### Cell lines and cell culture

All DT40 mutants were derived from isogenic parent DT40 cell lines (Additional file 1: **Table S1**). The DT40 cells and their mutant cells were cultured as a suspension in a humidified, 5% CO_2_ atmosphere at 39.5 °C. The medium consisted of RPMI-1640 cell culture medium containing 10% fetal bovine serum (heat inactivated), 1% chicken serum, 100 μg/ml penicillin and 100 μg/ml streptomycin. SUM149 (BRCA1-deficient breast cancer cell line, BRCA1 mutant c.2288delT, p.N723fsX13) [27], MCF12A (BRCA1-proficient normal breast epithelial cell line) [28], and isogenic RKO and *FANCC*^*−/−*^ colon cancer cell lines [16] were kindly provided by Drs. Stephen P. Ethier, William B. Coleman, and Scott E. Kern, respectively [16, 29, 30]. SUM149 and MCF12A cells were maintained in Ham’s F-12 medium with 5% fetal bovine serum, 5 μg/ml insulin, 2 μg/ml hydrocortisone, 5 μg/ml gentamicin, and 2.5 μg/ml fungizone. RKO and *FANCC*^*−/−*^ cells were cultured as reported previously [16]. The mammalian cells were cultured in a humidified, 5% CO_2_ atmosphere at 37 °C.

### Cell survival assay

Suspended cells (approximately 660 cells/250 ml) were seeded into 24-well plates, exposed to HMTA and allowed to divide for approximately 10 cell cycles. Cell proliferation for each plate was monitored under a microscope until the end of cultivation. When appropriate, LA was added to the culture immediately before exposure to HMTA. After cultivation, the cells were treated with XTT and the electron mediator 1-methoxy PMS. XTT, a yellow tetrazolium salt, is cleaved by mitochondrial dehydrogenase in metabolically active cells to form an orange formazan dye. A 96-well plate reader was used to measure the formazan dye at 450 nm with a 650 nm reference. Absorbance values for the exposed cells were then compared to those for the controls to obtain percent survival rates [16].

### Measurement of culture medium pH

To determine the effect of LA on culture medium pH, LA was added to complete RPMI-1640 medium in the absence of cells with subsequent incubation in a CO_2_ incubator as described above. A pH meter was then used to determine culture medium pH at 0, 10, 30 and 60 min after LA addition (Fig. 3a).

## Results and discussions

### Hypersensitivity of FANC/BRCA-deficient cells to HMTA in acidic conditions

**Fig. 2:**
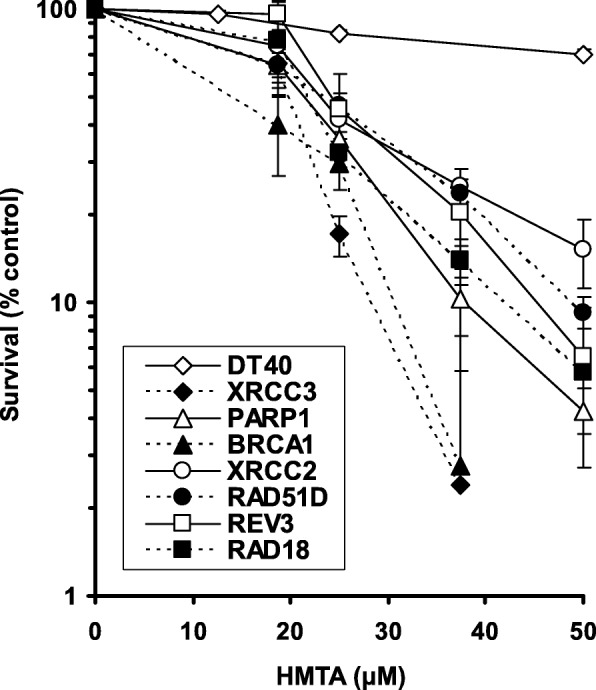
Sensitivity of DT40 cells and DT40-derived HR-deficient cells to HMTA. Survival curves of parental DT40 cells and HR-deficient DT40 mutant cells exposed to HMTA under neutral conditions. Cell survival of *wild-type* (*wt*) and DT40 mutant cells after exposure to HMTA at physiological pH

**Fig. 3:**
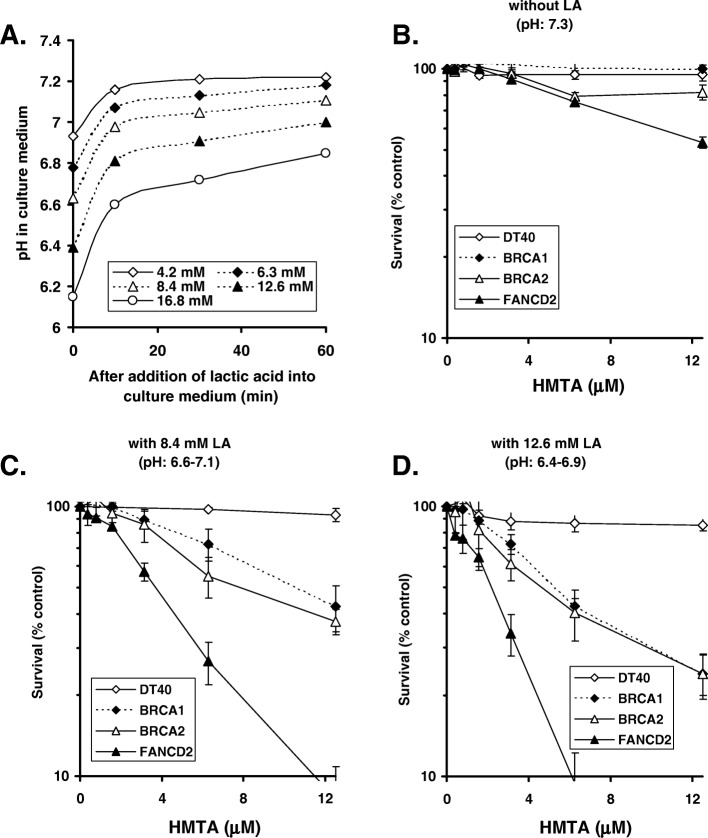
Sensitivity of DT40 cells and DT40-derived FANC/BRCA-deficient cells to HMTA. (**a**) Effects of lactic acid (LA) on the pH of complete medium at 39.5 °C in CO2 incubator. (**b**-**d**) Survival curves of parental DT40 cells and DT40 mutant cells exposed to HMTA at physiological pH (**b**), in the presence of 8.4 mM LA (pH 6.6–7.1) (**c**), and, in the presence of 12.6 mM LA (pH 6.4–6.9) (**d**)

**Fig. 4:**
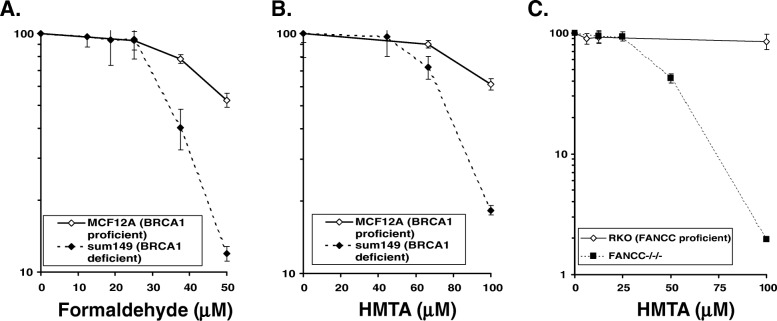
Sensitivity of human cultured cells proficient and deficient in HR to formaldehyde or HMTA. Survival curves of MCF12 (BRCA1-proficient) breast epithelial cells and and sum149 (BRCA1-deficient) breast cancer cells exposed to formaldehyde (**a**) or HMTA (**b**) under neutral conditions. (**c**) Survival curves of isogenic FancC-proficient or deficient RKO human colon cancer cells exposed to HMTA under neutral conditions

We addressed the cytotoxicity of HMTA to cells deficient in DNA repair under neutral and acidic conditions. Remarkably, although HMTA is not efficiently hydrolyzed under neutral conditions [20], all HR-deficient cells we tested were hypersensitive to HMTA in standard culture medium at pH 7.3 (Fig. 2). It has been reported that cancer cells frequently exist in an environment with the pH ranging from 6.4 to 6.8 [19]. Since HMTA is hydrolyzed more efficiently at pH 6.8 compared with an environment at neutral pH [20], we further investigated whether an acidic environment induced by LA increases toxicity of HMTA specifically in HR-deficient cells at low concentrations. In the presence of either 8.4 or 12.6 mM LA, HMTA clearly increased cell toxicity in FANCD2, BRCA1 and BRCA2 deficient cells (Fig. 3b-d), while there was no major sensitivity in parental DT40 cells to HMTA at pH 7.3 (no LA) (Fig. 3b). The cell toxicity induced by HMTA increased in a LA concentration-dependent manner in FANC/BRCA deficient cells. Conversely, a concentration of 12.5 μM or below of HMTA causes no sensitization in wt DT40 cells even in the presence of LA. These data indicate that acidic conditions potentiate the cell toxicity of HMTA specifically in FANC/BRCA deficient cells. Although FANC/BRCA deficient human cancer cells utilized in our study were sensitive to mild acidic conditions, we confirmed that either formaldehyde or HMTA causes hypersensitivity in BRCA1 deficient human breast cancer cells (Fig. 4a, b) as well as FANC deficient colon cancer cells (Fig. 4c) under neutral conditions. The different sensitivity to HMTA observed between MCF12 (BRCA1-proficient) cells and RKO (FANCC-proficient) cells could be due to the efficiency of formaldehyde detoxication by glutathione-dependent formaldehyde dehydrogenase.

### Advantage of HMTA for chemotherapy

Based on the in vivo studies performed by Masunaga’s group [23–26] and the present study, HMTA appears to selectively generate formaldehyde within a tumor mass thereby killing highly proliferating cancer cells as well as quiescent tumor cells without major adverse effects in mice. Furthermore, HMTA causes cell death for cells deficient in the FANC/BRCA pathway with higher selectivity under acidic conditions than under neutral environments. Taken together, we are currently proposing the mode of action of HMTA is to selectively kill FANC/BRCA deficient tumor cells with a dual targeting process as described below (Fig. 1). Under the mildly acidic conditions of the tumor environment, HMTA is hydrolyzed to formaldehyde and preferentially introduces DNA damage in cancer cells **(1st targeting: preferential delivery of formaldehyde to cancer cells).** Formaldehyde selectively kills BRCA/FANC pathway deficient or HR deficient cells, presumably due to deficiency in the repair of formaldehyde-induced DNA damage (**2nd targeting: selective cancer cell death)**. In contrast, normal cells are resistant to HMTA because less formaldehyde is formed in a neutral environment and they are proficient in the repair of formaldehyde-induced DNA damage. We believe HMTA can potentially be a dual-targeting cancer therapeutic.

HMTA may be effective as a dual-targeting chemotherapeutic agent in patients with breast and ovarian cancer with germ line mutations of *BRCA1* or *BRCA2* gene. HMTA can also be an attractive chemotherapeutic agent for sporadic cancers with reduced expression of proteins involved in the FANC/BRCA pathway as well as HR. It is worthwhile to note that there is a possible usage of HMTA for Fanconi anemia patients with cancer. Fanconi anemia is a rare autosomal or X-linked recessive disease associated with chromosomal instability, aplastic anemia, congenital abnormalities and a high risk of cancer, including acute myeloid leukemia and squamous cell carcinomas [8, 9, 12]. Due to defects in the repair of DNA damage, these patients have had poor tolerance for radiotherapy and chemotherapy, and the tumors were often aggressive. Therefore, it is essential to selectively target tumor cells in Fanconi anemia patients without damaging non-tumor cells. We believe HMTA could have the potential to cause cell death specifically for FANC deficient tumor cells in Fanconi anemia patients through our proposed dual-targeting mechanism.

A covalent linkage of doxorubicin to DNA leads to DNA interstrand crosslink mediated by formaldehyde [31]. In fact, several reports have touted the efficacy of doxazolidine and doxoform (doxorubicin/formaldehyde prodrugs) as being highly effective [32]. However, drugs such as doxazolidine/doxoform do not have the advantage of targeting tumors; therefore, doxazolidine/doxoform will quite likely have the same side-effects as doxorubicin, albeit less so. In contrast, the combination of HMTA and doxorubicin may well have very minimal such effects. Indeed, it has been reported that HMTA used in conjunction with the anthracycline antitumor drugs such as doxorubicin have a more powerful effect in a mouse tumor model [24]. We believe that the prophylactic use of HMTA may reduce the likelihood of cancer development in FANC/BRCA deficient people.

Finally, Patel’s group recently discovered that formate derived from endogenous formaldehyde with alcohol dehydrogenase 3 (ALD3/ALD5) were used as one-carbon metabolism for synthesizing DNA [33]. Methotrexate is a chemotherapy drug that blocks the folate-dependent one-carbon cycle by inhibition of dihydrofolate reductase leading to inhibition of DNA synthesis [34]. With methotrexate blocking of the one-carbon cycle, cancer cells could utilize formaldehyde-derived formate as a one carbon molecule source. Therefore, a combination of ALD3/ALD5 inhibitor and methotrexate may further decrease DNA synthesis in cancer cells. Furthermore, a combination of methotrexate, a formaldehyde catabolism inhibitor and HMTA could decrease DNA synthesis in cancer cells in addition to formaldehyde-induced toxicity particularly in cancer cells deficient in FANC/BRCA pathways.

## Conclusions

In the present study, we demonstrated that HMTA causes drastic cytotoxicity specifically in cultured cells deficient in the FANC/BRCA pathway at low pH. These results strongly suggest that HMTA may be an attractive, dual-targeting chemotherapeutic/preventive drug for the selective delivery of formaldehyde to solid tumors while causing cell death in FANC/BRCA-deficient cells without major adverse effects. In addition, a combination of HMTA and other chemotherapeutic drugs such as doxorubicin or methotrexate could be promising chemotherapeutic strategies for solid cancer deficient in FANC/BRCA pathways.

## Supplementary information

**Additional file 1: Table S1.** DNA repair genes mutated in the analyzed DT40 clones.

## Data Availability

The analyzed dataset and materials during the current study will be provided from the corresponding author on reasonable request.

## Abbreviations

1-methoxy PMS: 1-methoxy-5-methylphenazinium methyl sulfate
ALD3/ALD5: Alcohol dehydrogenase 3
DPC: DNA-protein cross-link
DSB: DNA double strand-break
HMTA: Hexamethylenetetramine
HR: Homologous recombination
ICLs: Interstrand cross-links
LA: Lactic acid
XTT: 2,3-bis (2-methoxy-4-nitro-5-sulfophenyl)-2H-tetrazolium-5-carboxanilide
