# Supplementary material for: Acid-specific formaldehyde donor is a potential, dual targeting cancer chemotherapeutic/chemo preventive drug for FANC/BRCA-mutant cancer
Source: Genes Environ. 2019 Dec 27;41:23. doi: 10.1186/s41021-019-0136-5 (PMC6921423; doi:10.1186/s41021-019-0136-5)
Supplement: Supplementary file 1 — Additional file 1: Table S1. DNA repair genes mutated in the analyzed DT40 clones. [file 41021_2019_136_MOESM1_ESM.ppt]

## Slide 1
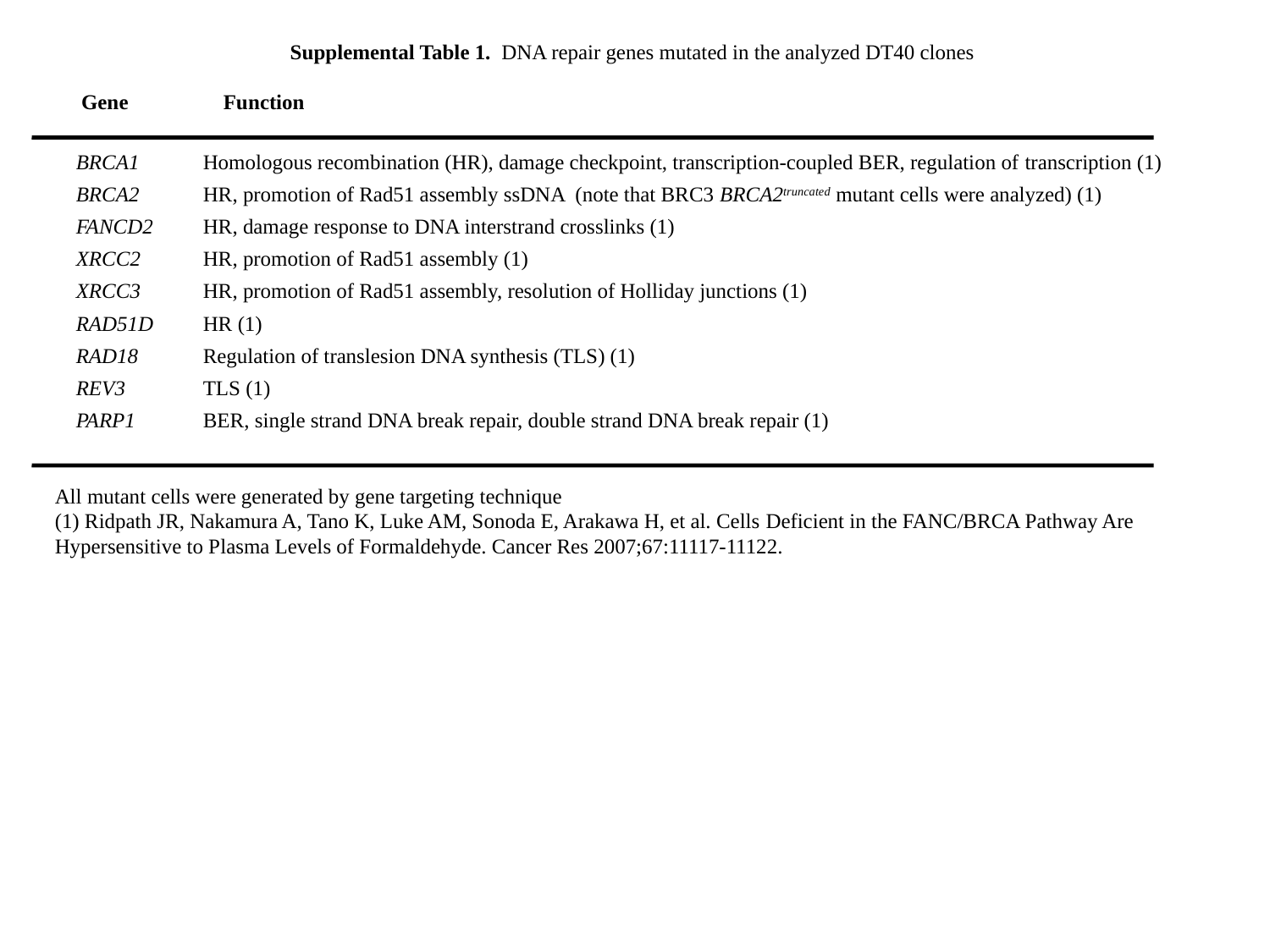

Supplemental Table 1. DNA repair genes mutated in the analyzed DT40 clones
 Gene Function
BRCA1 	Homologous recombination (HR), damage checkpoint, transcription-coupled BER, regulation of transcription (1)
BRCA2	HR, promotion of Rad51 assembly ssDNA (note that BRC3 BRCA2truncated mutant cells were analyzed) (1)
FANCD2	HR, damage response to DNA interstrand crosslinks (1)
XRCC2	HR, promotion of Rad51 assembly (1)
XRCC3	HR, promotion of Rad51 assembly, resolution of Holliday junctions (1)
RAD51D	HR (1)
RAD18 	Regulation of translesion DNA synthesis (TLS) (1)
REV3	TLS (1)
PARP1	BER, single strand DNA break repair, double strand DNA break repair (1)
All mutant cells were generated by gene targeting technique
(1) Ridpath JR, Nakamura A, Tano K, Luke AM, Sonoda E, Arakawa H, et al. Cells Deficient in the FANC/BRCA Pathway Are Hypersensitive to Plasma Levels of Formaldehyde. Cancer Res 2007;67:11117-11122.
